# Microarray-Based Comparative Genomic and Transcriptome Analysis of *Borrelia burgdorferi*

**DOI:** 10.3390/microarrays5020009

**Published:** 2016-04-16

**Authors:** Radha Iyer, Ira Schwartz

**Affiliations:** Department of Microbiology and Immunology, New York Medical College, School of Medicine, Valhalla, NY 10595, USA; radha_iyer@nymc.edu

**Keywords:** microarray, *Borrelia burgdorferi*, transcriptome, Lyme disease, transcriptional regulators

## Abstract

*Borrelia burgdorferi*, the spirochetal agent of Lyme disease, is maintained in nature in a cycle involving a tick vector and a mammalian host. Adaptation to the diverse conditions of temperature, pH, oxygen tension and nutrient availability in these two environments requires the precise orchestration of gene expression. Over 25 microarray analyses relating to *B. burgdorferi* genomics and transcriptomics have been published. The majority of these studies has explored the global transcriptome under a variety of conditions and has contributed substantially to the current understanding of *B. burgdorferi* transcriptional regulation. In this review, we present a summary of these studies with particular focus on those that helped define the roles of transcriptional regulators in modulating gene expression in the tick and mammalian milieus. By performing comparative analysis of results derived from the published microarray expression profiling studies, we identified composite gene lists comprising differentially expressed genes in these two environments. Further, we explored the overlap between the regulatory circuits that function during the tick and mammalian phases of the enzootic cycle. Taken together, the data indicate that there is interplay among the distinct signaling pathways that function in feeding ticks and during adaptation to growth in the mammal.

## 1. Introduction

The spirochete *Borrelia burgdorferi* is the causative agent of Lyme disease, the most commonly reported arthropod-borne disease in the United States [1,2,3]. *B. burgdorferi* is maintained in a natural enzootic cycle involving small mammals and a tick vector of the *Ixodes* species [3,4]. These two diverse host environments vary with respect to temperature, pH, oxygen tension and nutrients [5]. In order to adapt to growth in its mammalian and tick hosts, the spirochete must profoundly alter its gene expression in response to these environmental cues. *B. burgdorferi* has a unique genome organization; the genome of strain B31 (the type strain) is comprised of a linear chromosome of 910,724 bp and 21 linear and circular plasmids totaling an additional 610,694 bp [6,7]. Elucidation of the complete genome sequence enabled production of whole genome arrays. Since 2002, more than 25 microarray-based studies on the comparative genomic structure and transcriptome of *B. burgdorferi* have been published. Here, we review the contribution of microarray technology to our understanding of *B. burgdorferi* biology with particular emphasis on the variation in gene expression under different environmental conditions.

### B. burgdorferi Microarray Methodology

To date, all *B. burgdorferi* genome arrays have been designed based on the genome sequence of strain B31. Initially, whole genome arrays were constructed with PCR products of >1600 putative *B. burgdorferi* open reading frames (ORFs) and were spotted on either glass slides or nylon membranes [8,9,10]. Subsequently, 70-mer oligonucleotides spotted on glass slides were employed in order to improve the reliability of hybridization signal intensity [11,12]. In addition, several groups have employed other custom glass slide or chip designs representing the complete genome or smaller sub-arrays of selected ORFs [13,14,15]. Table 1 contains a listing of all published *B. burgdorferi* microarray studies and provides the array types employed.

In general, the steps for studying global gene expression changes in the *B. burgdorferi* transcriptome are as follows: RNA isolation, generation of labeled cDNA, array hybridization and scanning, data acquisition and analysis. In initial studies with nylon membrane arrays, cDNA was radioactively labeled with ^33^P; a detailed protocol is described in Ojaimi *et al.* [9]. Subsequently, a variety of high density microarray designs were developed and used fluorescently labeled DNA or cDNA for hybridization. Data acquisition, normalization and statistical analysis were particular to each type of microarray employed and details can be found in the respective references in Table 1. All published microarray data were deposited either in Array Express or NCBI’s Gene Expression Omnibus (GEO) databases. In addition, the results of virtually all published microarray studies reported to date have been validated by quantitative reverse transcription polymerase chain reaction (qRT-PCR).

## 2. Comparative Genomic Studies

Liang *et al.* [13], constructed a sub-array comprised of PCR products for 137 putative lipoproteins in order to study lipoprotein gene content across three *B. burgdorferi* sensu lato genospecies that cause Lyme disease in humans. There was extensive conservation of chromosomally-encoded lipoprotein gene content among all strains tested. By contrast, lipoproteins encoded on the plasmid portion of the genome were substantially less conserved [13]. This pattern was confirmed by Terekhova *et al.* [11], who employed whole genome microarrays to perform comparative genome hybridization of seventeen *B. burgdorferi* isolates, including clinical isolates with varying capacity for hematogenous dissemination in mice or humans. This revealed that chromosomal genes are more highly conserved among the isolates than are plasmid genes. The linear chromosome and plasmids lp54 and cp26 are the most conserved genomic elements among all isolates studied, which implies that they may encode functions required for bacterial viability. The most substantial variation was found among the linear plasmid portion of the genome; this variability was the result of presence/absence of entire plasmids, deletions or nucleotide sequence divergence.

Zhong and Barbour [16] used *B. burgdorferi* whole genome membrane arrays to study the similarity in gene content between *B. burgdorferi* and *B. hermsii*, a relapsing fever spirochete. They demonstrated that *B. hermsii* genomic DNA cross-hybridized with 81% of *B. burgdorferi* chromosomal genes and 46% of plasmid ORFs. They were also able to demonstrate the expression of 642 genes with similarity to *B. burgdorferi* ORFs in the blood of *B. hermsii*-infected mice [16].

Taken together, these microarray studies demonstrated that there is relatively little variation in the chromosomal portion of the *B. burgdorferi* genome, but much greater variation in plasmid content and sequence. Genomic sequencing of multiple *B. burgdorferi* isolates subsequently validated these findings [34,35,36].

## 3. Global Transcriptome Studies

The principal application of microarray technology to *B. burgdorferi* has been for global transcriptome analysis. These studies have informed our understanding of regulation of *B. burgdorferi* gene expression under different environmental conditions and, most importantly, elucidation of the roles for several transcriptional regulators in this process. As noted, in nature *B. burgdorferi* cycle through two distinct environments—tick vector and mammalian host. The limited number of organisms present in infected ticks or mammals constrains robust global transcriptome analysis from *in vivo* material. Initial transcriptome studies employed *in vitro* cultivation of *B. burgdorferi* in BSK medium under conditions thought to mimic either the tick or mammalian environments as surrogates for the *in vivo* state. Although subsequent studies demonstrated that global transcriptome analyses of *in vitro*-cultivated organisms do not fully reflect the *in vivo* state [12], these initial studies provided valuable insights into *B. burgdorferi* gene regulation.

### 3.1. Response to Temperature

To identify temperature-responsive genes, Ojaimi *et al.* [10] compared gene expression of *B. burgdorferi* cells grown at 23 or 35 °C (to mimic the tick or mammalian environment, respectively). 215 genes were differentially expressed at the two temperatures; with 133 showing greater expression at 35 °C relative to 23 °C. Interestingly, 136/215 (63%) temperature-responsive genes were encoded on plasmids. Of particular note, are linear plasmid lp54 and the circular cp32 plasmids; 45% of the putative ORFs encoded on lp54 exhibited temperature-regulated expression and >20% of cp32-encoded ORFs responded to temperature shift. Transcripts known to have elevated levels during mammalian infection (e.g., those for outer surface protein C (OspC), decorin binding proteins A/B (DbpAB) and the alternative sigma factor RpoS) displayed elevated expression at 35 °C. Similarly, genes subsequently shown to be more highly expressed during the tick phase (glycerol uptake and utilization operon (*glp*FKD) and *chb*C, encoding a component of the chitobiose transporter) had significantly elevated transcript levels at 23 °C [10].

Revel *et al.* [8] carried out a similar analysis, but also varied the pH of the growth medium so as to mimic the environment in the unfed tick (23 °C/pH 7.5) and fed tick states (37 °C/pH 6.8). A total of 94 genes were differentially expressed between the two temperatures; 79 had higher expression at 37 °C. Among transcripts elevated at the higher temperature were those for OspC and DbpA/B, as expected. In addition, transcripts for chemotaxis and motility functions and the OppA components of the oligopeptide ABC transporter were also elevated under the “fed tick” condition. Fifteen transcripts encoded on lp54 were differentially expressed, consistent with the findings of Ojaimi *et al.* [10]. Interestingly, there was only limited concordance between the Ojaimi and Revel data sets. This is likely the result of methodological differences between the two studies, including different array types (membrane array *vs.* glass slide microarray), pH of the BSK growth medium, slightly different temperatures (35 °C *vs.* 37 °C) and differences in the data analysis approaches.

### 3.2. Transcriptome of B. burgdorferi in the Host-Adapted State

The paucibacillary nature of *B. burgdorferi* infection in mammals led Akins *et al.* [37] to develop an alternative approach for isolating spirochetes in the host-adapted state. The method involves cultivating *B. burgdorferi* in BSK medium contained within dialysis membrane chambers (DMCs) implanted in a rat peritoneal cavity [37]. Numerous studies have demonstrated that gene expression of *B. burgdorferi* cultivated in DMCs is markedly different from that observed for spirochetes cultivated *in vitro* in the same medium at 37 °C [38,39,40]. Three microarray studies have been published in which transcriptome comparisons between *B. burgdorferi* cultivated *in vitro* at 37 °C and in DMCs were reported. In Revel *et al.* [8], 66 genes showed altered expression between these two conditions; only 6/66 exhibited higher expression in DMCs. Surprisingly, expression of some recognized mammalian phase genes such as *ospC* and *dbpA/B* was not induced. In a subsequent study by Brooks *et al.* [18], a total of 125 transcripts were differentially expressed between *B. burgdorferi* cultivated at 37 °C *in vitro* and DMCs—58 transcripts were induced (including *ospC*) and 67 were repressed. Among the latter, only three were chromosomally-encoded and the vast majority encode putative proteins of unknown function [18]. Interestingly, there was less than 10% overlap between the Revel and Brooks’ datasets, likely the result of methodological differences between the two studies. Caimano *et al.* [12], also performed whole transcriptome analysis of *B. burgdorferi* grown at 37 °C and in DMCs. Their study was designed to identify the regulon controlled by RpoS and a direct comparison of wild-type *B. burgdorferi* at the two conditions was not provided. However, the results clearly demonstrate that gene expression differs substantially between *in vitro*- and DMC-cultivated organisms. Taken together, these findings demonstrate that temperature alone does not elicit the distinctive mammalian host modulation of *B. burgdorferi* gene expression, but in addition requires mammalian host-specific signals.

As an alternative to DMC cultivation, Tokarz *et al.* [19], examined the combined effect of temperature and blood so as to simulate the environmental changes *B. burgdorferi* encounter as they transit from tick vector to a mammalian host. Spirochetes were incubated in the presence or absence of 6% human blood for 48 h and transcriptomes were compared. A total of 154 transcripts were differentially expressed in the presence of blood (75 induced and 79 repressed relative to no addition of blood); greater than two-thirds of the regulated transcripts are plasmid-encoded. Among the induced transcripts were those for OspC and DbpA, as expected, transcripts encoding for chemotaxis and motility functions and for two transcriptional regulators, RpoS and BosR.

Given the induction of RpoS by incubation with human blood, it was of interest to compare the list of differentially expressed genes during blood co-incubation to that of RpoS-regulated genes [12]. Thirty-nine genes were found in common; 36/39 are activated by RpoS during *in vitro* cultivation at 37 °C and during co-incubation with blood. Further, 40 genes that were differentially expressed in the presence of human blood were also RpoS-regulated during growth in DMCs (28 induced, 12 repressed). This analysis indicates that RpoS is induced during the nymphal blood meal and controls a regulon required for tick-to-mammal transmission and during mammalian infection and is supported by additional microarray studies discussed below.

Livengood *et al.* [14], performed global transcriptome analysis of *B. burgdorferi* following a 20-h incubation with human neuroglial cells. A total of 72 *B. burgdorferi* transcripts were differentially expressed in the neuroglial cells relative to *in vitro*-cultivated spirochetes; the levels of 58 were induced and 14 were repressed. 63/72 differentially expressed genes are located on either the chromosome or plasmid lp54. Numerous genes involved in motility/chemotaxis were induced in neuroglial cells, as was *ospC*. Among the transcripts with decreased expression was *glpK*, which has been shown in other studies to be repressed in the mammalian environment [41,33].

### 3.3. Transcriptome of B. burgdorferi in the Tick Vector

Iyer *et al.* [33], characterized and compared the transcriptional profiles of *B. burgdorferi* during acquisition (fed larvae), transmission (fed nymphs) and in a mammalian host-like environment (DMCs). This analysis required the introduction of a pre-amplification step prior to array hybridization in order to enrich for *B. burgdorferi* RNA [33]. A core transcriptome consisting of 397 genes was expressed under all experimental conditions and is likely required for spirochetal survival in nature. The three *in vivo* transcriptomes differ substantially among each other, as well as to that obtained from organisms cultivated *in vitro* at 37 °C indicating that spirochetes respond to a variety of host-specific signals. Among the key findings were the differential expression of genes encoding lipoproteins, transporters and enzymes in several metabolic pathways including the oxidative branch of the pentose phosphate pathway, glycerophospholipid biosynthesis and isoprenoid biosynthesis. Alterations in gene expression for chemotaxis/motility proteins were also noted suggesting that the chemotaxis/motility apparatus may vary in the tick and mammalian environments. This was the first report describing *B. burgdorferi* global gene expression profiles from *in vivo* samples containing limited copies of pathogen. The findings provide the necessary transcriptional framework for delineating *B. burgdorferi* regulatory pathways that operate throughout the enzootic cycle.

## 4. Transcriptional Regulation

As already noted, *B. burgdorferi* must alter its gene expression program in order to adapt to growth in either the tick or mammalian environments. This adaptation is mediated by several transcriptional regulators including RpoS, Rrp1, BosR and Rel_Bbu_ [4,5]. RpoS, an alternative sigma factor, controls a regulon whose members are important for transmission of *B. burgdorferi* from tick vector to mammalian host and/or during mammalian infection. Expression of RpoS is controlled by a signaling cascade involving Rrp2, a response regulator, and RpoN, a second alternative sigma factor [4,5]. Another signaling pathway comprised of Hk1 and Rrp1 promotes the synthesis of cyclic di-GMP and expression of c-di-GMP-dependent genes; evidence indicates that genes comprising this regulon are required for spirochetal survival in ticks [27,42]. In addition, BosR and Rel_Bbu_ have been shown to control expression of substantial numbers of genes [22,28,43].

Microarray analyses have informed much of our current understanding of transcriptional regulation in *B. burgdorferi*. Comparative transcriptome studies employing regulatory mutants have been particularly helpful in defining the regulons controlled by various transcriptional regulators. In this section, we review these studies and also provide a secondary analysis by merging the statistically significant gene lists from the processed data reported in comparisons of wild type and mutant transcriptomes for components of the Rrp2-RpoN-RpoS and Hk1-Rrp1 regulatory cascades. In addition, we also included transcriptome data for BosR in these analyses.

### 4.1. Rrp2-RpoN-RpoS Regulatory Cascade

Caimano *et al.* [12], performed a comparative microarray analysis of *B. burgdorferi* strain 297 wild-type and an *rpoS* mutant cultivated either *in vitro* following temperature-shift to 37 °C or within DMCs. The expression of 110 genes was affected by the absence of RpoS during *in vitro* growth; all had higher expression in the wild type strain implying that their transcription is at least partially dependent on RpoS. No transcripts were found to be significantly repressed. 137 genes had altered expression in spirochetes cultivated under mammalian host-like conditions (*i.e.*, in DMCs); 103 transcripts had significantly elevated levels in wild type relative to the RpoS mutant and 44 of these were also higher *in vitro*. Importantly, in contrast to *in vitro*-grown spirochetes, 34 genes had higher expression in mutant *B. burgdorferi* cultivated in DMCs demonstrating that host-specific signals are required for RpoS-dependent repression. Significantly, a number of genes in this group (*ospA*, *bba62*, *glp* operon) have been shown to have higher transcript levels in ticks [40,33].

Norgard and co-workers generated individual mutants in *rrp2*, *rpoN* and *rpoS* in a strain 297 background and compared gene expression between wild-type and mutant strains *in vitro* [24]. They identified 98 genes that were regulated in common by either Rrp2, RpoN or RpoS; 97 exhibited higher expression in wild type and only one (*bba62*) had lower expression. The substantial overlap between genes regulated by RpoS and RpoN provides evidence that the two alternative sigma factors form a congruous pathway and that RpoN regulates *B. burgdorferi* gene expression through RpoS [44,45]. Importantly, two-thirds (68/98) of the genes were similarly regulated by RpoS in the study by Caimano *et al.* [12]. It is noteworthy that transcription of an additional 106 genes was affected in the *rrp2* mutant. This implies that Rrp2 controls expression of a regulon unrelated to the RpoS response.

Two additional publications reported on the RpoS, RpoN and Rrp2 regulons. Boardman *et al.* [23] generated an *rrp2* mutant in an infectious strain B31 background. They observed 144 genes with altered expression in the mutant. Due to strain variation, the overlap among the two Rrp2 gene sets was only 42%. Fisher *et al.* [25], studied the RpoS and RpoN regulons using mutants in each of these alternative sigma factors. Curiously, there is <20% concordance between these datasets and those of Caimano *et al.* [12], Ouyang *et al.* [24] and Boardman *et al.* [23] probably the result of methodological differences.

To generate a composite picture of the Rrp2-RpoN-RpoS regulatory circuit, we merged the datasets from Caimano *et al.* [12], Ouyang *et al.* [24], and Boardman *et al.* [23]. The composite gene list contained 395 genes. This gene set (referred as rrp2-RpoN-RpoS) is provided in Table S1 and was used for further analysis as described below.

### 4.2. Borrelia Oxidative Stress Regulator (BosR)

Borrelia oxidative stress regulator (BosR; BB0647) was initially thought to mediate the oxidative stress response in *B. burgdorferi* [46,47]. Subsequently, it was shown to be required for transcription of RpoS [22,48,49]. Two groups have generated *bosR* mutants and performed microarray-based comparative transcriptome analyses. Hyde *et al.* [21], employed a BosR point mutant that was sensitive to oxidative stress and an insertional disruption of this point mutant that restored resistance to oxidative stress. Due to the unusual nature of the strains used (both comparison strains were mutants and no wild-type strain was included), this study is not considered further. Ouyang *et al.* [22], inactivated *bosR* in an infectious B31 strain background. They found the BosR regulon to encompass 199 genes, 137 of which were induced. These induced genes included *rpoS* and *ospC*, as was expected, and nearly two-thirds (87/137) were also part of the RpoS regulon described by these investigators [24].

**Figure 1:**
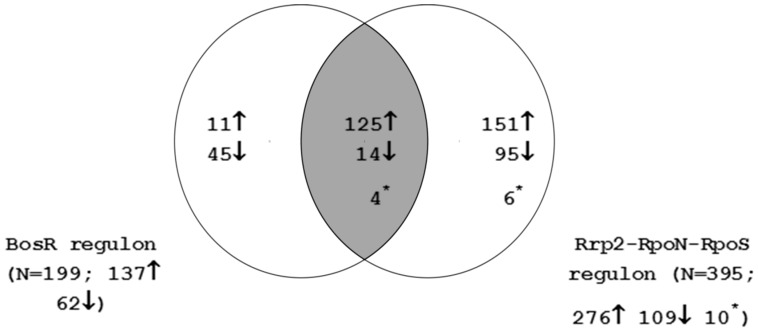
Overlap between the Rrp2-RpoN-RpoS and BosR regulons. Numbers indicate the count of genes that show statistically significant induction (up arrow) or repression (down arrow). Asterisk indicates genes that are induced in one regulon and repressed in the other.

The genes regulated by the Rrp2-RpoN-RpoS cascade were compared to those in the BosR regulon as shown in Figure 1. The Venn diagram reveals a substantial overlap of Rrp2-RpoN-RpoS activated genes to those activated by BosR (125/137; 91%). This finding supports the accumulating evidence that both RpoS and BosR are required for modulation of gene expression during mammalian infection [22,49,50,51]. For further analysis (see below), the Rrp2-RpoN-RpoS and BosR regulated genes were merged to generate an Rrp2-RpoN-RpoS-BosR regulon consisting of 451 genes. The additional 56 genes that are regulated by BosR only are given in Table S2. This gene set should represent *B. burgdorferi* genes that are differentially regulated during late nymphal tick feeding, tick-to-mammal transmission and during mammalian infection.

### 4.3. Hk1-Rrp1 Regulatory Circuit

Three groups have reported the construction of *rrp1* mutants and studied their gene expression profiles compared to those of the parent strain. Rogers *et al.* [26], generated an *rrp1* mutant in a non-infectious strain background that also lacked many plasmids. They identified 140 transcripts with altered abundance, 131 of which had higher expression in the wild-type than the mutant. These included products of the *glp* operon, *bba74* and *spoVG* which have been shown to be repressed by RpoS in DMCs and expressed at higher levels in ticks [12,33,40]. Subsequently, He *et al.* [27] and Caimano *et al.* [42] employed mutants that were generated in an infectious strain B31 background; both mutants can infect mice but cannot survive in ticks. He *et al.* [27] found that 120 genes had altered expression; 99 had higher transcript levels in the wild type strain. Although Caimano *et al.* [42], used RNA-seq, these two datasets were merged to generate a composite *rrp1* regulon. The regulon contained a total of 297 genes (222 induced and 75 repressed) (Table S3).

### 4.4. Interaction between the Rrp2-RpoN-RpoS-BosR and Hk1-Rrp1 Regulatory Circuits

**Figure 2:**
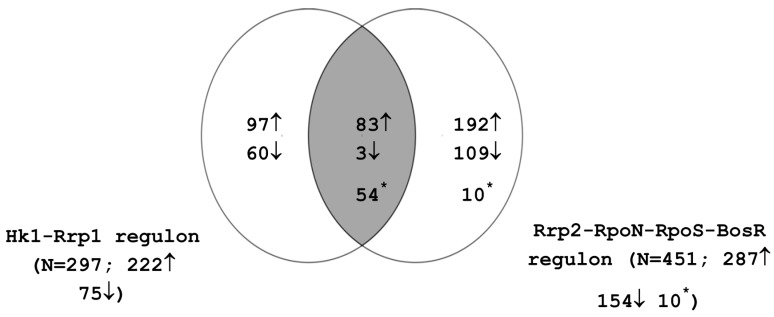
Interplay between the Rrp2-RpoN-RpoS-BosR and Hk1-Rrp1 regulons. Numbers indicate the count of genes that show statistically significant induction (up arrow) or repression (down arrow). Asterisk indicates genes that are induced in one regulon and repressed in the other.

Regulation of differential gene expression during the enzootic cycle is mediated primarily by RpoS and Rrp1. RpoS is responsible for modulating gene expression during spirochetal transmission from the tick vector to the mammalian host and during mammalian infection; BosR also plays a role in these processes. Rrp1 mediates changes in tick phase gene expression and regulates protective responses during the tick blood meal [4,27,42]. The interplay of the two regulatory circuits controlling RpoS and Rrp1 activity is thus critical to the adaptation and survival of *B. burgdorferi* in the vector and host milieus. The overlap between these two regulatory cascades has not been well characterized. In order to gain further insight into the linkage between these pathways, we compared the genes comprising the Rrp2-RpoN-RpoS-BosR regulon (Tables S1 and S2) with those comprising the Hk1-Rrp1 regulon (Table S3). The resulting Venn diagram is presented in Figure 2. 140 genes were regulated by both pathways; 83 genes were activated by both Hk1-Rrp1 and Rrp2-RpoN-RpoS-BosR and three genes were commonly repressed by both pathways. In addition, 42 genes were activated by Hk1-Rrp1 but repressed by Rrp2-RpoN-RpoS-BosR and 12 genes were induced by the RpoS pathway but repressed by Rrp1. Interestingly, the 42 genes induced by Rrp1 and repressed by RpoS include the *glp* operon, *spoVG*, *bba62* and *bba74*. These genes are known to have significantly higher expression in ticks than in the mammalian host [33,40,41,42]. These findings are consistent with the model that Rrp1 controls a regulon whose members are required during the tick phase of the *B. burgdorferi* life cycle [4,42].

## 5. Conclusions and Prospects for the Future

Microarray studies have contributed significantly to the current understanding of *B. burgdorferi* genome content and transcriptional regulation. Delineation of differential gene expression patterns throughout the enzootic cycle and characterization of the regulons controlled by various transcriptional regulators mediating these processes have provided roadmaps for more detailed mechanistic investigations. The limitations of microarray analyses include the inability to detect low copy transcripts and small RNAs, restriction of gene/transcript detection to only those genes represented on the microarray and failure to recognize post-transcriptional processing events. NextGen sequencing methodologies are not subject to these limitations and will ultimately replace microarray approaches for comparative genomic and transcriptomic investigations.

## Figures and Tables

**Table 1:** Published studies utilizing *B. burgdorferi* microarrays.

Experimental Condition	Strain	Microarray Type	Reference
Comparative genomics	B31	Glass slide	Liang *et al.*, *Infect. Immun.*, 2002 [13]
Comparative genomics	B31	membrane	Zhong & Barbour, *Mol. Microbiol.*, 2004 [16]
Comparative genomics	B31	70 m oligo glass slide	Terekhova *et al.*, *J. Bacteriol.*, 2006 [11]
Temperature response	B31	Membrane	Ojaimi *et al.*, *Infect. Immun.*, 2003 [10]
Strain transcriptome comparison	B31	Membrane	Ojaimi *et al.*, *Infect. Immun.*, 2005 [17]
*In vitro* and host-adapted (DMC)	B31	Glass slide	Revel *et al.*, *PNAS*, 2002 [8]
*In vitro* and host-adapted (DMC)	B31	Membrane	Brooks *et al.*, *Infect. Immun.*, 2003 [18]
Blood co-incubation	B31	Membrane	Tokarz *et al.*, *Infect. Immun.*, 2004 [19]
Monoclonal OspB antibody co-cultivation	B31	Membrane	Anderton *et al.*, *Infect. Immun.*, 2004 [20]
Neuroglial cell co-incubation	B31	Affymetrix slide	Livengood *et al.*, *Infect. Immun.*, 2008 [14]
RpoS regulon	297	70 m oligo glass slide	Caimano *et al.*, *Mol. Microbiol.*, 2007 [12]
BosR regulon	B31	Membrane array	Hyde *et al.*, *Microbiology*, 2006 [21]
BosR regulon	B31	70 m oligo glass slide	Ouyang *et al.*, *Mol. Microbiol.*, 2009 [22]
Rrp2 regulon	B31	70 m oligo glass slide	Boardman *et al.*, *Infect. Immun.*, 2008 [23]
Rrp2/RpoN/RpoS regulon	297	70 m oligo glass slide	Ouyang *et al.*, *Microbiology*, 2008 [24]
RpoN/RpoS regulon	B31	70 m oligo glass slide	Fisher *et al.*, *PNAS*, 2005 [25]
Rrp1 regulon	B31	70 m oligo glass slide	Rogers *et al.*, *Mol. Microbiol.*, 2009 [26]
Rrp1 regulon	B31	70 m oligo glass slide	He *et al.*, *PLoS Pathog.*, 2011 [27]
Rel_Bbu_ regulon	297	70 m oligo glass slide	Bugrysheva *et al.*, *PLoS ONE*, 2015 [28]
BadR regulon	B31	Nimblegen	Miller *et al.*, *Mol. Microbiol.*, 2013 [15]
HrpA regulon	B31	Nimblegen	Salman-Dilgimen *et al.*, *PLoS Pathog.*, 2013 [29]
Non-human primate tissues	N40, JD1	Glass slide	Narasimhan *et al.*, *PNAS*, 2003 [30]
Fed Ticks	N40	Glass slide	Narasimhan *et al.*, *J. Bacteriol.*, 2002 [31]
Mouse tissues	B31	70 m oligo glass slide	Pal *et al.*, *J. Infect. Dis.*, 2008 [32]
Tick feeding stages and host-adapted (DMC)	B31	70 m oligo glass slide	Iyer *et al.*, *Mol. Microbiol.*, 2015 [33]

## Supplementary Materials

The following are available online at http://www.mdpi.com/2076-3905/5/2/9/s1, Table S1: *B. burgdorferi* genes regulated by Rrp2-RpoN-RpoS as identified by whole genome microarray analysis; Table S2: *B. burgdorferi* genes regulated by BosR only as identified by whole genome microarray analysis; Table S3: *B. burgdorferi* genes regulated by Hk1-Rrp1 as identified by global transcriptome analysis.
